# Further Characterization of *HDAC* and *SIRT* Gene Expression Patterns in Pancreatic Cancer and Their Relation to Disease Outcome

**DOI:** 10.1371/journal.pone.0108520

**Published:** 2014-10-02

**Authors:** Mehdi Ouaïssi, Françoise Silvy, Céline Loncle, Diva Ferraz da Silva, Carla Martins Abreu, Emmanuelle Martinez, Patrick Berthézene, Sophie Cadra, Yves Patrice Le Treut, Jean Hardwigsen, Bernard Sastre, Igor Sielezneff, Liliane Benkoel, Jean Delgrande, Ali Ouaissi, Juan Iovanna, Dominique Lombardo, Eric Mas

**Affiliations:** 1 Aix-Marseille University, CRO2, UMR_S 911, Marseille, France; 2 INSERM UMR 911, Marseille, France; 3 AP-HM, Timone Hospital, Department of Digestive and visceral Surgery, Marseille, France; 4 Faculdade de Farmácia da Universidade do Porto, Porto, Portugal; 5 Aix-Marseille University, CRCM, UMR_S 1068, Institut Paoli-Calmettes, CNRS, UMR7258, Marseille, France; 6 INSERM, UMR 1068, Marseille, France; 7 AP-HM, La Conception Hospital, Department of hepatic transplantation and general surgery, Marseille, France; 8 Aix-Marseille University, Marseille, France; 9 AP-HM, Timone Hospital, Department of histopathology, Marseille, France; Peking University Health Science Center, China

## Abstract

Ductal adenocarcinoma of the pancreas is ranking 4 for patient' death from malignant disease in Western countries, with no satisfactory treatment. We re-examined more precisely the histone deacetylases (*HDAC*) and Sirtuin (*SIRT*) gene expression patterns in pancreatic cancer with more pancreatic tumors and normal tissues. We also examinedthe possible relationship between *HDAC* gene expression levels and long term disease outcome. Moreover, we have evaluated by using an *in vitro* model system of human pancreatic tumor cell line whether HDAC7 knockdown may affect the cell behavior. We analyzed 29 pancreatic adenocarcinoma (PA), 9 chronic pancreatitis (CP), 8 benign pancreatic (BP) and 11 normal pancreatic tissues. Concerning pancreatic adenocarcinoma, we were able to collect biopsies at the tumor periphery. To assess the possible involvement of HDAC7 in cell proliferation capacity, we have generated recombinant human Panc-1 tumor which underexpressed or overexpressed HDAC7. The expression of *HDAC1,2,3,4,7 and Nur77* increased in PA samples at levels significantly higher than those observed in the CP group (*p* = 0.0160; 0.0114; 0.0227; 0.0440; 0.0136; 0.0004, respectively). The expression of HDAC7, was significantly greater in the PA compared with BP tissue samples (*p* = 0.05). Mean mRNA transcription levels of PA for *HDAC7* and *HDAC2* were higher when compared to their counterpart biopsies taken at the tumor periphery (*p* = 0.0346, 0.0053, respectively). Moreover, the data obtained using confocal microscopy and a quantitative method of immunofluorescence staining strongly support the HDAC7 overexpression in PA surgical specimens. The number of deaths and recurrences at the end of follow up were significantly greater in patients with overexpression of *HDAC7*. Interestingly, the rate of growth was significantly reduced in the case of cell carrying shRNA construct targeting HDAC7 encoding gene when compared to the parental Panc-1 tumor cells (p = 0.0015) at 48 h and 96 h (p = 0.0021). This study strongly support the notion that HDAC7play a role in pancreatic adenocarcinoma progression.

## Introduction

Ductal adenocarcinoma of the pancreas is ranking 4 for patient' death from malignant disease in Western countries [1]. The aggressiveness of this cancer is demonstrated by a disease-related mortality rate closely approximating the incidence [2]. Cancer diffusion and metastasis account for approximately 90% of all cancer related deaths [2]. Metastasis follows a multi-step complex processes in which neighboring healthy tissue is invaded by primary tumor cells, which access the systemic circulation and finally proliferate at distant sites into macroscopic secondary tumors via the perivascular and/or perilymphatic tissue [3]. In the case of pancreatic cancer, most of the patients already have metastases at the time of diagnosis. A number of investigations have focused on the identification of possible markers that may allow for early diagnosis of pancreatic cancer. Specific events that promote tumorigenesis and cancer progression are linked with complex molecular modifications such as DNA methylation, histone acetylation, phosphorylation, ubiquitylation and ADP ribosylation. Currently, results from basic research underline the importance of acetylation and deacetylation at the level of not only histone lysine residues but also other cellular factors that are supposed to interfere with the regulation of gene expression. In fact, the steady-sate of acetylation of core histones is controlled by the opposing actions histone actetyltransferases (HATs) and histone deacetylases (HDACs) whose activities are correlated with gene activation and gene repression or silencing [4]. Growing knowledge about HDACs shows that they are regulators of growth, differentiation and cell death (apoptosis). The dysfunction of transcriptional repression mediated by HDACs may lead to carcinogenesis. Indeed, modulation of expression levels of genes encoding HDACs (over- and/or under-expression) has been reported for different types of cancer [5], [6], [7], [8]. The characterization of key genes that play a role in pancreatic tumor development may not only allow to uncover new biomarkers, which will become the focus of intensive research interest, but will also shed light on potential gene products to be exploited for the design of selective means to interfere with tumor progression. Recent studies demonstrated [9], [10] that HDAC2 was overexpressed in pancreatic adenocarcinoma tissue samples. In order to provide insight into the biological behavior of pancreatic cancer and identify new potential biomarkers, we have in the past few years initiated a study aiming to examine the levels of *HDAC* and *SIRT* genes expression in a set of surgically resected pancreatic tissues including 11 pancreatic adenocarcinoma samples and a normal pancreas tissue. Despite relatively small number of specimens examined, we found increased expression of HDAC7, a class IIa deacetylase, in 9 out of 11 samples [11], [12]. However, although we have used one normal pancreatic as the calibrator for gene expression measurement and other samples in close proximity or far away from the tumor as controls, we thought that further investigations using a greater number of normal pancreatic tissues and tumor samples are needed to re-examine more precisely the *HDAC* and *SIRTs* gene expression patterns in pancreatic cancer. Moreover, attempts were made to examine the possible relationship between HDAC gene expression and the disease outcome. We also evaluate by using an *in vitro* model system of human pancreatic tumor cell line whether HDAC7 knockdown may affect the cell behavior.

## Materials and Methods

### Subject population

From May 2007 through August 2012, 29 pancreatic adenocarcinoma (PA), 9 chronic pancreatitis (CP), 8 benign pancreatic tumors including serous cystadenoma (SC) n = 2, mucinous cystadenoma (n = 2), benign IMPN (n = 2), benign cyst (retentional cyst, n = 1), and pancreatic endocrine tumor (n = 1), were taken in charge in the department of Surgery at la Timone Hospital (Marseille, France). All patients underwent contrast-enhanced thoracic and abdominal computed tomography, abdominal ultrasonography, magnetic resonance imaging and blood testing. PA had no preoperative treatment before surgery. Twenty two pancreaticoduodenectomies (PD), and 7 left pancreatectomies were conducted for pancreatic adenocarcinoma, respectively. Two PD, 4SP and 3 Frey procedures [13]were performed for CP. Four PD, 2 SP and 2 medial pancreatectomies were performed for benign lesions. Four normal pancreatic (NP) biopsies were obtained during liver transplantation on the donor hepatectomy, 7others were obtained during susmesocolic surgery when radical gastrectomy required left pancreatectomy: 3 ampulloma (AP1-3), 2 cholangiocarcinoma of the principal bile duct (BD1-2), 1 gastrinoma of the duodenum (G), 1 normal adjacent tissues samples after gastric resection for gastric adenocarcinoma. In addition, 11 samples of control tissues taken at the periphery of the surgical specimens from different patients with PA were also included in this study. Data were prospectively collected and a standardized questionnaire was completed at the time of follow up and of study assessment. Prior to surgery all patients had signed an informed consent form that had been approved by the local ethics committee. Protection committee people of South Mediterranean II, approved by Ministerial Order dated May 31, 2012, constituted under the order of the Director General of Health Agency Region Provence Alpes Côte d'Azur dated 13 June 2012, composed of: L. BOYER, V. PRADEL, B. DUSSOL, C. SICHEL, M. CAILLOL, F. VINCENT, G. NAURAYE, JP. VIDAL, O. SCHWEITZER, J. ACCIARO, discussed in plenary the declaration file storage and preparation for scientific elements of the human body identified by the Ministry of Higher Education and Research under the reference DC-2013-1857 and whose scientific director is Mr Dominique LOMBARDO, gave a favorable expert advice (Files S1, S2).

### Surgery

All surgical procedures were performed by three experienced pancreatic surgeons (BS, YPLT, IS, MO). Experienced senior surgeons carried out all pancreatic head resections. PD was performed using a pylorus preserving or Whipple procedure, and an end-to-end pancreaticojejunostomy (PJA) was constructed with a single-layer anastomosis of interrupted 5–0 PDS (Ethicon surgery) absorbable sutures. Choice of surgical technique was decided per-operatively bound to surgeon's decision. Anterior SMA approach was then routinely used since the year 2001 to standardize the radicality of resection at the site of the retroperitoneal margin. Standard lymphadenectomy was performed before the year 2001 and extended lymphadenectomy since that time [14]. Frozen section examinations at the pancreatic trans-section line, was performed in all cases and was not invaded for all PA. Standard lymphadenectomy was carried out along the hepatoduodenal ligament and the common hepatic artery [15]. All resections were performed via laparotomy. In the case of left pancreatectomy: the technique of distal pancreatectomy beginning with division of pancreatic neck before control of splenic vessels was used [16]. Early neck division allows safer vascular control. For distal pancreatectomy, primary section of the neck and splenic vessels ligation, combined with division of left gastro-epiploic and short gastric vessels, precedes mobilization of a devascularized specimen, decreases operative bleeding and seems most suited from a carcinologic point of view. After surgery patients received adjuvant chemotherapy in function of their performance status, and at the discretion of the oncologist. Two soft drains (Peters) or left pancreatic section for the left pancreatectomy were routinely placed near the pancreaticojejunal anastomose. Operative time was recorded. In the absence of a fistula, drains were removed after 7 days.

### Tissue samples

All surgical specimens were reviewed by a senior pathologist. Clinical and pathologic staging (Table S1) were reassessed according to American Joint Committee on Cancer TNM staging of pancreatic cancer concerning PA. The tumors had been snap-frozen in RNA later and liquid nitrogen during 15 seconds and immediately stored at −80°C. Tumors characteristics were recorded in all patients (Table S1). This included its location, median size at diagnosis, UICC staging, extension of the neoplastic disease at diagnosis, number of metastasis sites when present and the nodal status. All specimens were graded according to the classification rules of the 6^th^ edition (2002) of the American Joint Committee on Cancer Staging Manuel (AJCC) [17]. The radicality of resection was graded according to R-classification of the International Union against Cancer (R0: no residual tumor, R1: microscopic residual tumor, R2: macroscopic residual tumor *in situ*) [18]. The retroperitoneal margin was graded R1 if residual microscopic tumor was identified within 1 mm of the trans-section line [19]. Since the year 2002, the pathological examination of the surgical specimen was standardized according to the Luttges *et al.* protocol [20], by using routine ink marking of the retroperitoneal trans-section line. In cases of vascular resection, the complete vascular segment was embedded and both ends were examined separately as additional resection margins.

### Tissue Treatment and immunofluorescence histological Study

Tissue specimens (21 PA and 6 NP samples) were routinely fixed in 10% formalin, embedded in paraffin and further cut into 5 mm sections immediately stored at 4°C or stained with hematoxylin-phloxine-saffron (HPS).

### Reagents and mAbs

In the case of Western blot analysis, a mouse monoclonal anti-actin antibody and POD-labelled anti-mouse antibody {from Sigma (St Louis, MO)}, were used. A rabbit polyclonal anti-HDAC7 antibody was from Euromedex (Souffelweyersheim, France), POD-labelled anti-rabbit antibody was from Cell Signaling (Beverly, MA). DMEM cell culture media, penicillin, streptomycin, trypsin-EDTA, hygromycin B and neomycin were from InVitrogen(Carlsbad, NM)To conduct the immunostaining, mouse monoclonal antibody (mAb) anti-HDAC7 (20 µg/mL, Sigma-Aldrich, France), and a rabbit anti-Nur77 antibody (Ab) (5 µg/mL, Thermo Scientific, CergyPontoise, France) were used. Biotin-conjugated F(ab')2 fragment of goat antibodies to mouse IgG (Beckman Coulter, Roissy CDG, France) or biotin-conjugated goat anti-rabbit IgG (Sigma-Aldrich) for HDAC7 and Nur77 immunostaining respectively were used.

### Immunofluorescence

Formalin-fixed, paraffin-embedded tissue sections (5 µm) were deparaffinized and treated with an antigen retrieval solution. Tissue sections were incubated 2 h at room temperature (RT) with anti-HDAC7 or anti-Nur77 and washed in PBS. Sections were then washed in PBS and incubated 1 h at RT with 1∶50 dilution of biotin-conjugated F(ab')2 fragment of goat antibodies to mouse IgG or biotin-conjugated goat anti-rabbit IgG for HDAC7 and Nur77 immunostaining respectively. The sections were washed in PBS, treated 1 h at RT with 1∶50 dilution of streptavidin-fluorescein (Beckman Coulter). All sections were mounted in Dako aqueous permanent mounting medium.

Sections were observed by means of a Zeiss Axiovert 200 M inverted microscope with 20 objective and a confocal laser scanning microscope (CLSM) (Leica, TCS SP5) with a 60 objective. An argon laser with an excitation of 488 nm was used to activate the green fluorescence.

### Image Processing

Images were processed as previously described [11]. For each primary antibody, the staining was calculated as the ratio between the total fluorescence of the area (total specific fluorescence) and the surface of this area (mean specific fluorescence, MSF). Mean values of six stained areas for each biopsy were then calculated.

### Follow up

Postoperative follow-up includes clinical, biochemical, and radiological assessment every 3 months during the first postoperative year, then, every 6 months up to a postoperative delay of 5 years and afterward every year up to 10 years of follow-up. None patient was lost of follow up. The surviving patients were assessed for disease recurrence and site of recurrence. Follow-up information was obtained from medical records and direct patients' consultation. Follow up was continued for all patients in this cohort to June 2013 without including new patients. Long-term follow-up was available for all patients. The mean duration of follow-up was 16 months (median: 18 months, range: 2–53). The length of survival was calculated from the date of the operation until the date of death or the date which the present study was ended.

### Cell growth and proliferation

Panc-1 cells (ATCC, CRL-1469) originated from human pancreatic carcinoma were grown in DMEM medium supplemented with 10% FCS, 1% penicillin (100 U/ml), 1% streptomycin (100 µg/ml) and glutamine (1 mM). Cells were seeded at 4000 cells *per* well in a 96-wells plate and cell growth was assessed at 24, 48, 72 and 96 h by 3-(4,5-dimethythiazol-2-yl)-2,5-diphenyl (MTT) assay. Results are given as mean ±SD (n = 8) and are representative of at least 3 independent experiments.

### Cells transfection

Silencing of HDAC7 gene was performed by stable transfection of Panc-1 cells with SureSilencing shRNA Plasmid for Human HDAC7 (Qiagen, Courtaboeuf, France), which confers hygromycin resistance to transfected cells. Over expression of HDAC7 was performed by stable transfection using pCDNA3-HDAC7-Flag plasmid (plasmid Addgene 13824; Eric Verdin, The J. David Gladstone Institutes, San Francisco, CA, [21]), which confers neomycin resistance to transfected cells. Panc-1 cells at 50-80% confluence were transfected with Lipofectamine LTX Reagent with PLUS Reagent (InVitrogen) according to the manufacturer' instructions. After a 6 h incubation at 37°C in 5% CO2, the transfection medium was replaced with complete DMEM medium for 48 h and then with fresh medium containing 300 µg/ml hygromycin B (shRNA plasmid) or 2 mg/ml neomycin (pCDNA3-HDAC7-Flag plasmid). After 1–2weeks, in selective medium a limit dilution was performed. Selected clones were referred to as SH or pFLAG cell clones, respectively. Control transfections were carried out using shRNA CTL vector or pCDNA3 empty vector.

### SDS-PAGE and Western blotting

Cells were washed three times with ice-cold PBS, harvested and pelleted by centrifugation. Pellets were washed twice and lysed at 4°C in 0.1 ml of lysis buffer {50 mM Tris/HCl pH 7.5, 100 mM NaCl, 0.5% Triton X-100, 1 mM EDTA, protease inhibitors (Complete TM, Roche Diagnostics)}. Homogenates were incubated for 30 min at 4°C and clarified by centrifugation at 10 000 g for 30 min at 4°C and frozen at −20°C. An aliquot was saved for protein determination using the Bicinchoninic Acid Kit (Sigma, St Louis). Equal amounts of cell lysates (100 µg) in reducing SDS buffer were resolved on gradient 8–16% Tris-Glycine polyacrylamide gels (Pierce). After electrophoretic migration, proteins were transferred onto nitrocellulose membranes and processed for immunoblotting by using appropriate primary and secondary antibodies. After washes, membranes were revealed by chemiluminescence (Roche diagnostic, Meylan, France).

### RNA isolation and reverse transcription

The tissues (≈30 mg) were disrupted in 600 µl Buffer RLT Plus (Qiagen) using an adapted sized vessel for disruption and homogenization with a Tissue Ruptor. Total RNA was isolated using the All Prep DNA/RNA mini kit (Qiagen) according to the manufacturer's instructions. RNA concentration was determined by absorption and RNA integrity was checked on RNA Nano chips (Agilent, Santa Clara, CA). Reverse transcription (RT) reactions were performed on 1 µg of total RNA using Improm-II Kit (Promega, Madison, WI) according to the manufacturer's instructions. T he cells were harvested and pellets for RNA purification were processed immediately in RLT lysis buffer (Qiagen). Total RNA was isolated using the RNeasy mini kit (Qiagen) according to the manufacturer's instructions. RNA samples were treated with DNase I (DNA-free kit, Ambion Inc., Austin, Texas) to remove traces of contaminant genomic DNA. Reverse transcription (RT) reactions were performed on 1 µg of total RNA using random hexamers and the M-MLV reverse transcriptase (Invitrogen) according to the manufacturer's instructions.

### Real-time quantitative PCR (Q RT-PCR)

**Figure 1:**
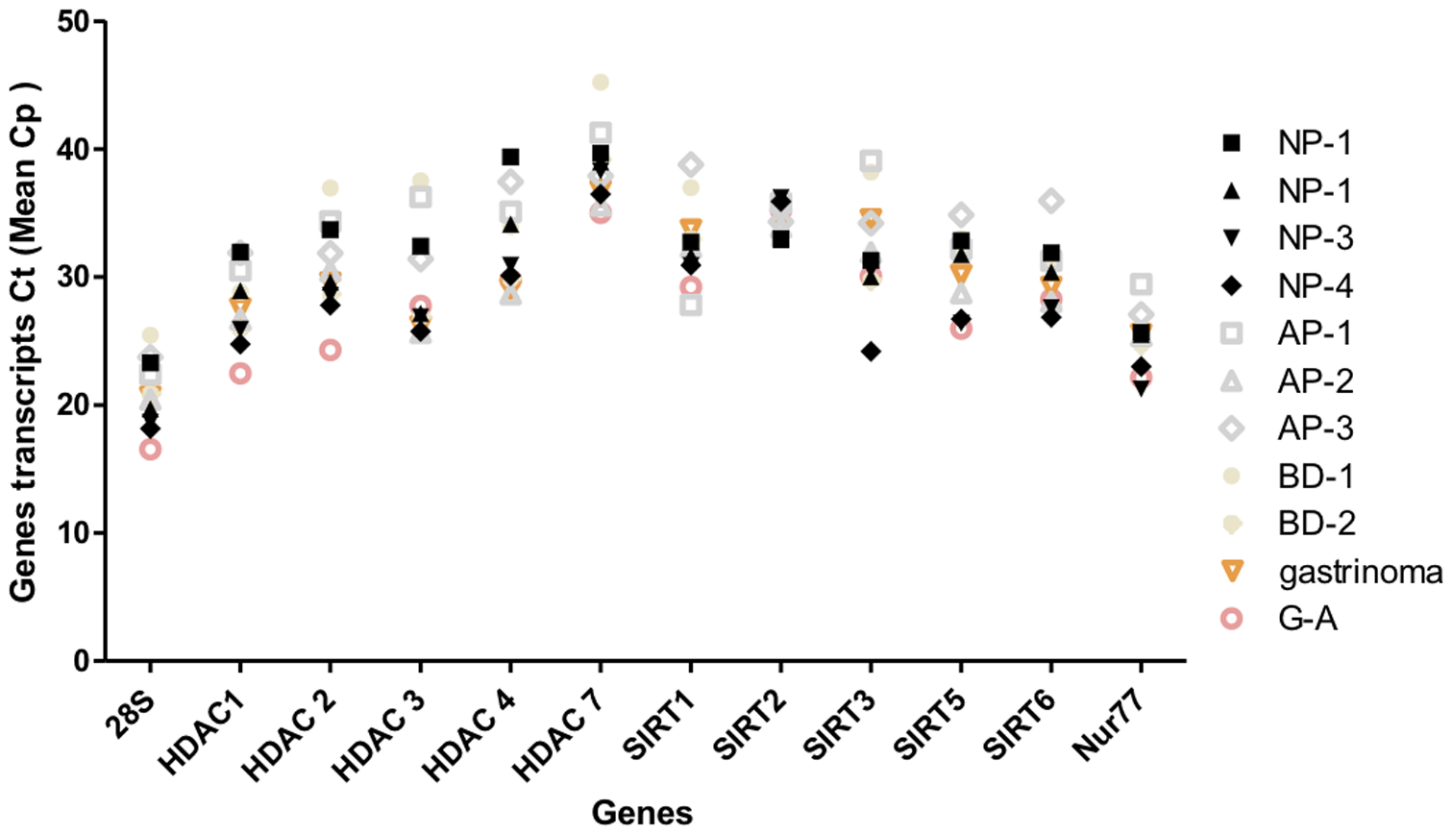
Quantitative real-time Q RT-PCR. Mean Cp of HDACs, SIRTs and Nurr77 genes and 28S transcripts of tissues samples from the control group. qPCR were run in triplicates on two independent cDNA preparations from pancreatic tissues as described in Materials and Methods section. The mean Cp values Ct (mean of Cp) were determined for the following samples: NP-1 to 4, normal pancreas; BD-1 and 2, normal pancreas samples from patients carrying biliary duct tumors. AP-1 to 3: normal adjacent tissues samples ampulloma; G-A: normal adjacent tissues samples after gastric resection for gastric adenocarcinoma; Gastrinoma: normal adjacent tissues samples for gastrinoma of the duodenum.

Q RT-PCR reactions were run in triplicate on two independent RNA preparations from cellular clones using the LightCycler480 SYBR Green I Master mix and the LightCycler real time PCR instrument (Roche Molecular Biochemicals, Mannheim, Germany) as described previously [12]. The primers are summarized in Table S2. Primers were designed to amplify an approximately 200 bp fragment in the coding sequence of 11 genes belonging to the HDAC/SIR families. The 28S RN gene was chosen as control. Comparative analysis of Crossing Point Ct values (mean of *Cp*) of the 4 NP and the 7 normal pancreatic biopsies for the set of HDAC and SIRT as well as Nur77 genes revealed that they were of similar values with no statistically significant difference (Figure 1, Table S3). Therefore, the 11 biopsies samples were designed as a control group (CG) and their mean *Cp* values for all genes were determined and used as the calibrator. The 2^−ΔΔCt^ method was used to analyze the relative gene expression [22]. The average Ct (Cp values) was calculated for both the target and the 28S gene and the ΔCt (C _t,*target*_-C _t,*28S*_) was determined. The CG was used as the calibrator [for calculation of ΔΔC_t_ =  (C _t,*target*_-C _t, *28S*_)-(C _t,*target CG*_-C _t,*28S CG*_)] [22]. For the CG, ΔΔC_t_ equals zero and 2^0^ equals one, so that the fold change in gene expression relative to the CG equals one. Evaluation of 2^−ΔΔCt^ indicates the fold change in gene expression relative to the CG.

### Statistical analysis

Statistical analysis was performed using Graph Pad 5 software. Data are expressed as mean ± the standard deviation or median with interquartile range. The differences between two groups were analyzed using the Mann-Whitney U test or student test t. One-way analysis of variance or Kruskal-wallis test was performed to compare more than two groups.

To compare for categorical variables, the chi-square test or Fisher exact was used. Kaplan-Meier method was used to estimate overall and relapse-free survival. For all tests, a p-value of less than 0.05 was considered significant.

## Results

### Expression of HDAC, SIRT and Nurr77 in control pancreatic tissues

Panel of 11 normal pancreas surgical specimens were studied. These tissues samples were evaluated for HDACs, SIRTs and Nur77 mRNA status by quantitative real-time PCR. As shown in Figure 1 and Table S3) when considering the crossing point (*Cp*) which defines the point at which the fluorescence rises appreciably above the back ground fluorescence, the values observed for each gene were not statistically significantly different (the p values varied from 0.13 to 1) suggesting that the target genes were expressed at similar levels in the surgical specimens examined. This allowed us to define the 11 surgical specimens as a control group (CG), therefore using the mean values of their *Cps* as the calibrator.

### Comparison of Q RT-PCR analysis of HDACs, SIRTs and Nur77 expressionbetween pancreatic cancer, chronic pancreatitis, benign tumor and control group

**Figure 2:**
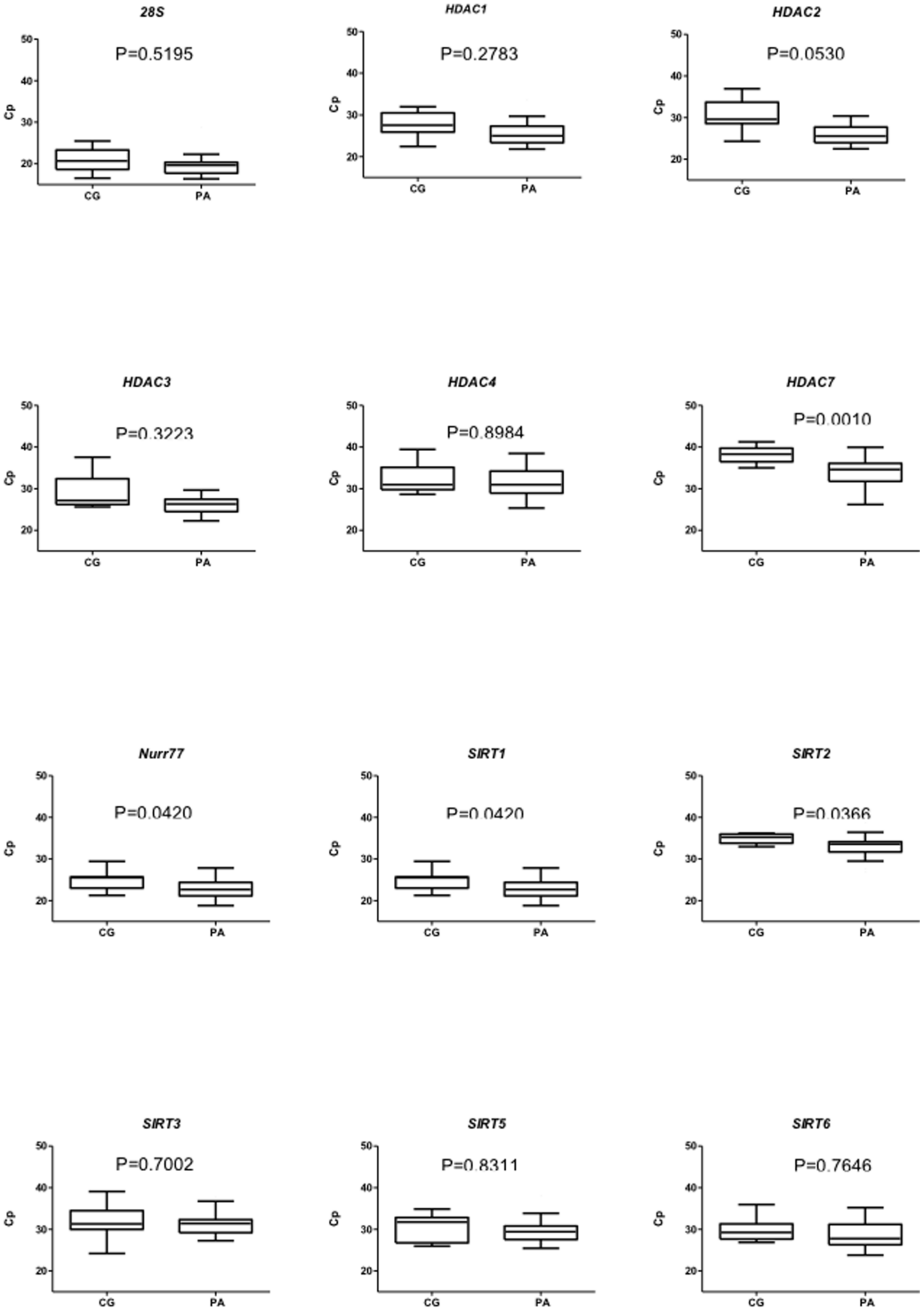
Expression of HDACs, SIRTs and Nurr77 in pancreatic adenocarcinoma (PA) and control group (CG) by Q RT-PCR. Samples were run in triplicate on two independent cDNA preparations from pancreatic tissues as described in Materials and Methods section. (Cp) Crossing point values. Comparisons were made by Wilcoxon test and differences were considered significant at p<0.05.

In our previous report [12] we have shown that HDAC7 is significantly expressed in PA tissues samples. Given that only 11 PA tissue specimens and one normal pancreatic biopsy were used, we performed qPCR analysis of *HDACs*, *SIRTs* and *Nur77* expression in a total of 46 tissues samples comprising 29 PA. Moreover, 11 normal pancreatic tissues biopsies were used as the calibrator to define more accurately the observable mRNA gene expression levels among the tissues from PA, CP and B. Mean *Cp* values (Ct) of *HDAC7*, *Nurr77*, *SIRT1*, *SIRT2*, were significantly lower in PA group compared to the CG (*p* = 0.0010; 0.040; 0.0420; 0.0366, respectively) (Figure 2). This result demonstrated that *HDAC7*, *Nurr77*, *SIRT1*, *SIRT2* were significantly over-expressed in PA samples than in the CG ones.

**Figure 3:**
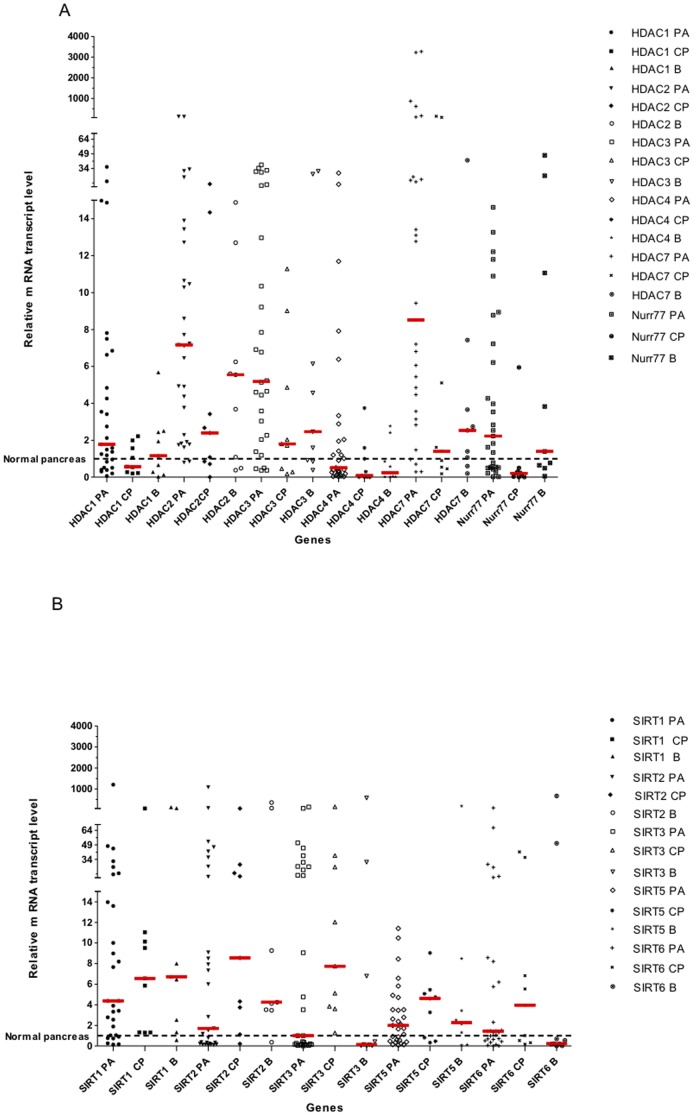
(A) HDACs and Nurr77 genes expression in pancreatic tumor tissues. (B)SIRT genes expression in pancreatic tumor tissues. Tissue samples from Pancreatic Adenocarcinoma (PA), Benin Tumor (B) and Chronic Pancreatitis (CP). Red line: median of relative RNA transcript levels.

We then applied the 2^−ΔΔCt^ method to analyze the relative gene expression in tissue samples from PA, B and PC [22]. As shown in Figure 3A, the expression of *HDAC1,2,3,4,7 and Nur77* increased in PA samples at levels significantly higher than those observed in the case of CP group (*p* = 0.0160; 0.0114; 0.0227; 0.0440; 0.0136; 0.0004, respectively). Moreover, the expression of HDAC7, was significantly higher in the PA compared to B tissue samples (*p* = 0.05).

Q RT-PCR analysis showed the expression of various SIRTs mRNAs in tissues from PA, CP, and B (Figure 3B). Although some significant variation at the level of *SIRT*s gene transcription could be evidenced, the data did not allow to identify a *SIRT* gene expression levels variation between PA, CP and B samples.

**Figure 4:**
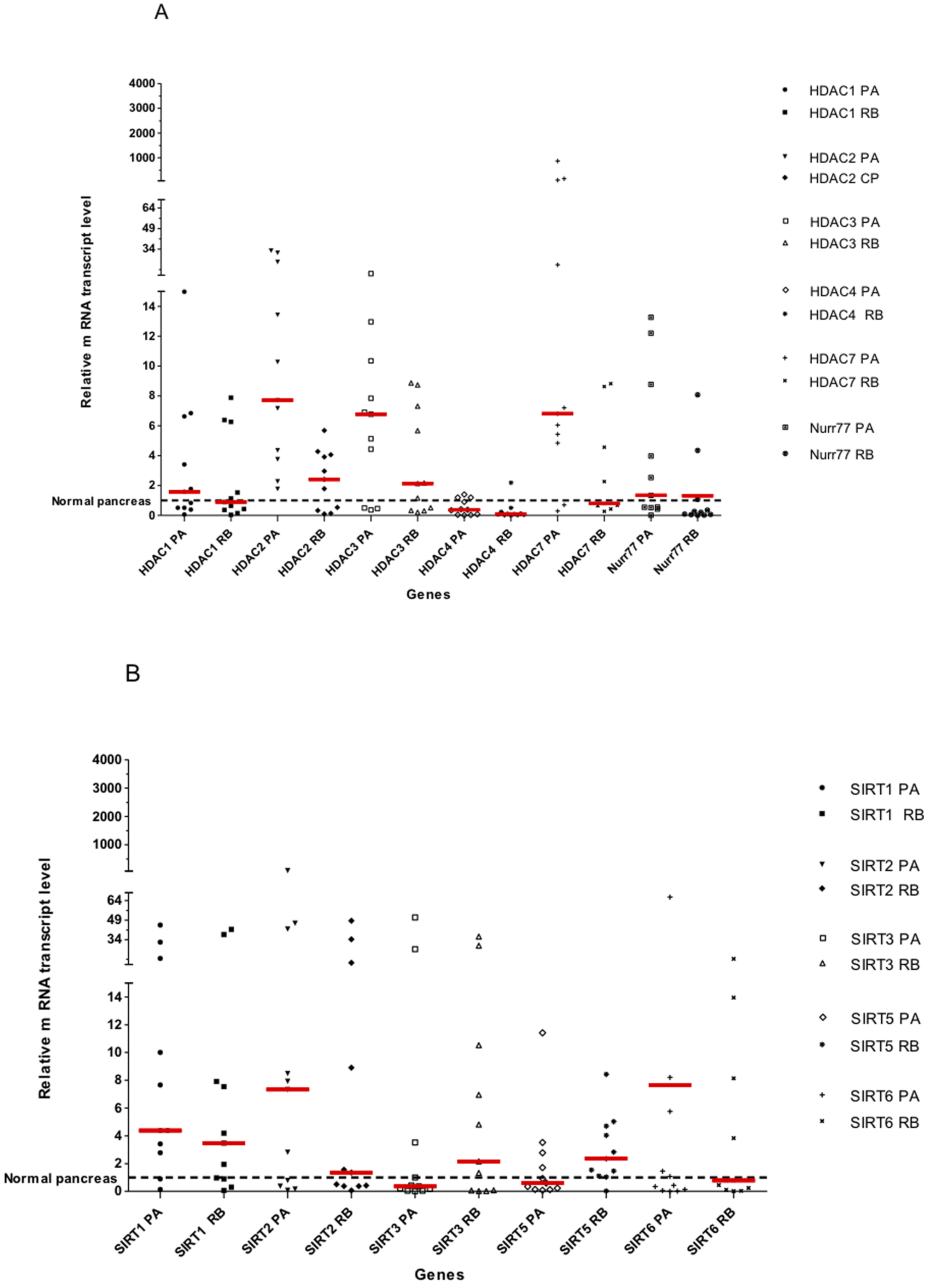
HDACs and Nurr77 gene expression (A) and SIRTs gene expression (B). Pancreatic adenocarcinoma tumors (PA), remote biopsies tissues (RB).

We further compared the levels of gene expression in some specimens where we were able to collect biopsies at the tumor periphery. As shown in Figure 4A, PA samples showed statistically higher mean mRNA transcription levels for *HDAC7* and *HDAC2*when compared to their counterpart biopsies taken at the tumor periphery (*p* = 0.0346, 0.0053, respectively). Although some degree of variation in *SIRTs* gene expression levels could be evidenced between tissue samples, no such remarkable difference as those observed for *HDAC7* and *HDAC2* could be seen. These data support the notion that among the *HDACs* and *SIRTs* gene examined, *HDAC7* and *HDAC2* are two genes with the highest potential to be markers of PA.

### Patterns and expression levels of HDAC7 and Nur77

**Figure 5:**
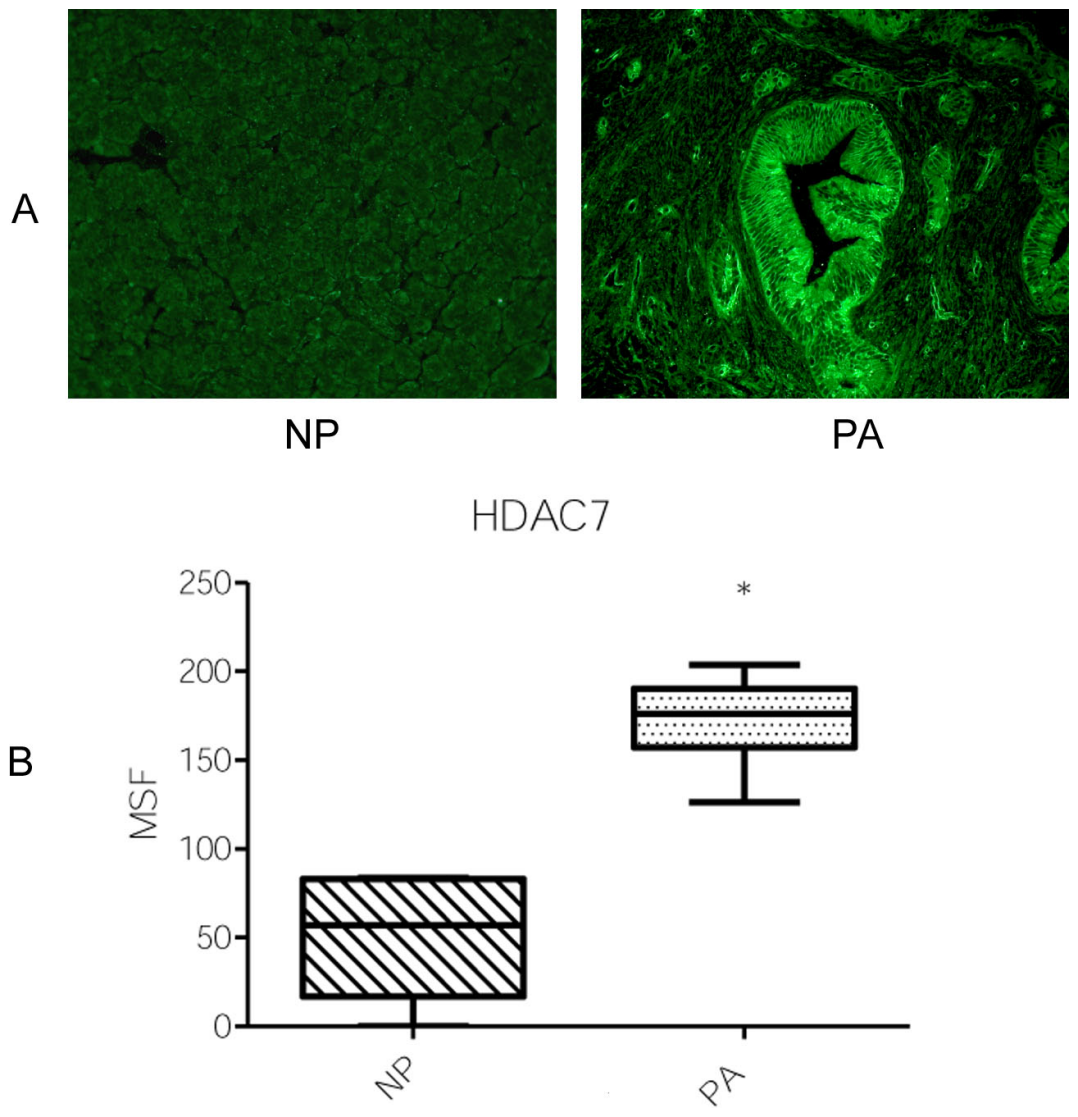
Representative results of immunofluorescence staining with HDAC7 mAb (A). A slight staining is found in NP. In PA, a strong staining is found in the cytoplasm and in association with the cell plasma membrane. Original magnification 250x. Quantitative determination of mean specific fluorescence (MSF) **(B)**. Six areas in each case were measured. Medians of the MSF intensities obtained with PA and NP tissues are represented by the horizontal lines and the interquartile range is represented by boxes. (* *P*<0.0004).

**Figure 6:**
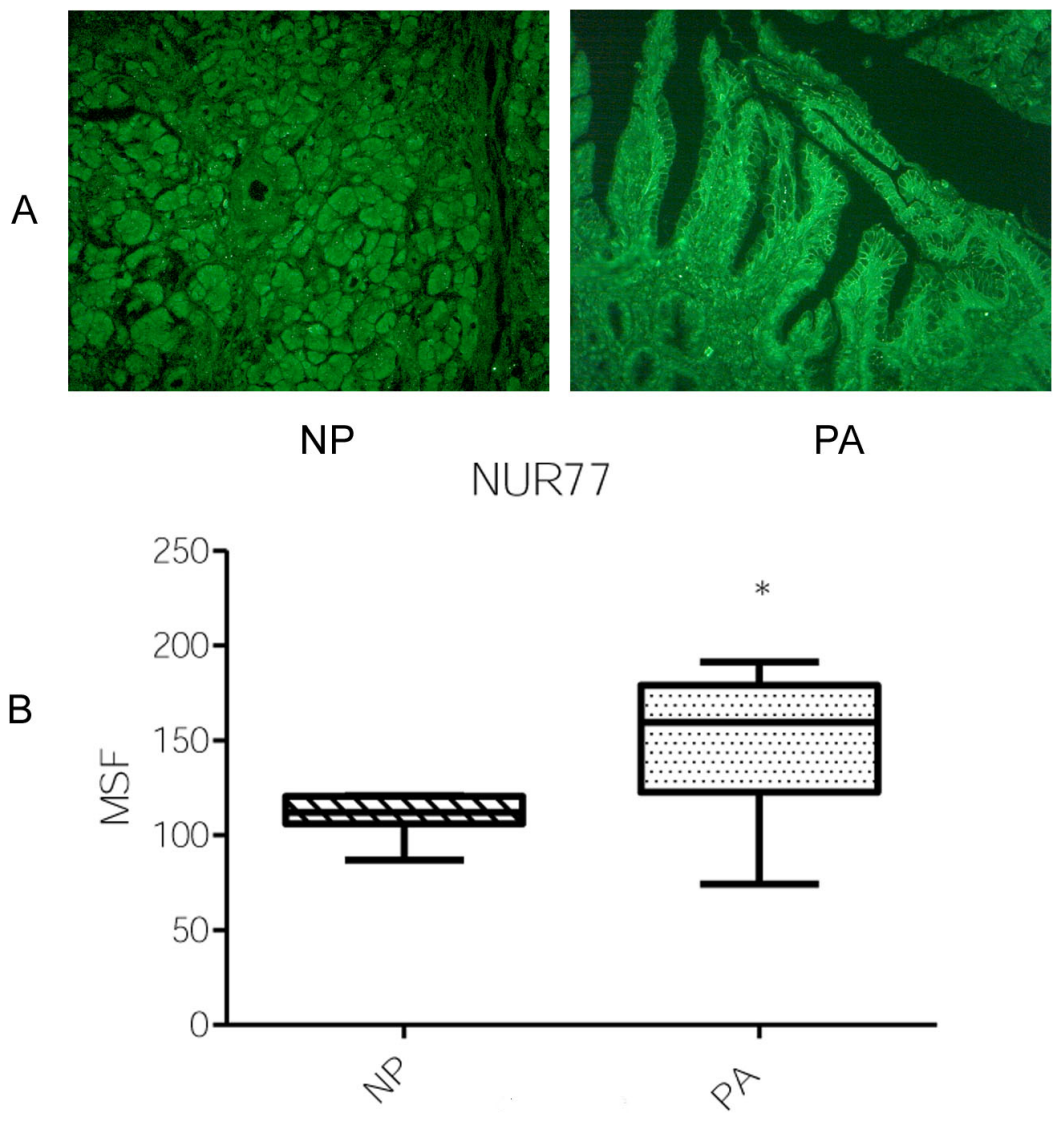
Representative results of immunofluorescence staining with Nur77 Ab (A). A moderate staining is found in the cytoplasm of NP. PA cells are strongly stained in the cytoplasm and a moderate staining was observed on the cell plasma membranes. Original magnification 250x. Quantitative determination of mean specific fluorescence (MSF) **(B)**. Six areas in each case were measured. Medians of the MSF intensities obtained with PA and NP tissues are represented by the horizontal lines and the interquartile range is represented by boxes. (* *P*<0.0225).

When using HDAC7 mAb, the staining was negative or slightly positive in control cases (NP), whereas strong positive immune reactivity was found in all PA (Figure 5A). To analyze more accurately the level of immunostaining in tissues samples, six stained areas for each immunofluorescence image were quantified by measuring the MSF intensity of stained areas. All PA exhibited statistically higher MSF values than control specimens. The average of the MSF intensity found with mAb to HDAC7 was significantly increased in PA (mean  = 173.78±4.46) compared to the control samples (mean  = 54.31±13.26) (Figure 5B) (*P* = 0.0004). Analysis of six stained areas for each immunofluorescence image by measuring the MSF intensity showed that the average of the MSF values found with mAb to Nur77 was significantly increased in PA (mean  = 146.50±8.07) compared to the control samples (mean  = 110.00±4.41) (Figure 6) (*P* = 0.0225).

### Impact of *HDAC7, HDAC2 and Nurr77* expression in outcome of patient with pancreatic adenocarcinoma

**Figure 7:**
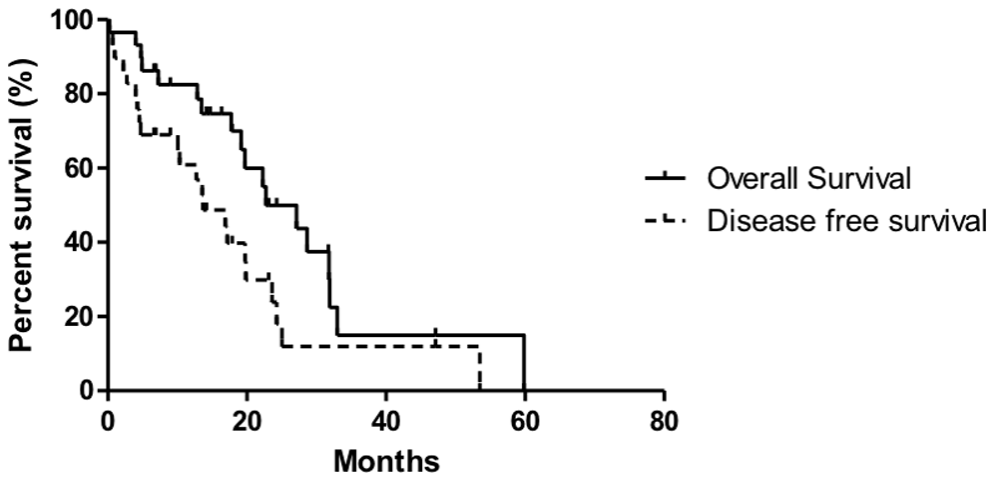
Overall and disease free survival of 29 patients with pancreatic adenocarcinoma. Overall (solid line) and disease-free survival (dashed line).

**Figure 8:**
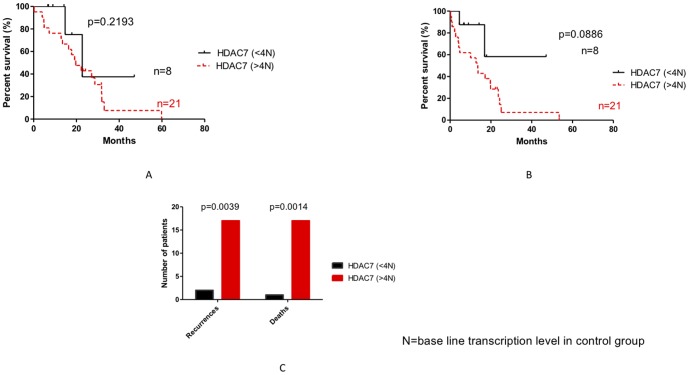
Impact of HDAC7 expression on the overall survival (A), the disease free survival (B) and recurrences and death in patients with pancreatic adenocarcinoma (C). N: base line transcription level in CG.

**Figure 9:**
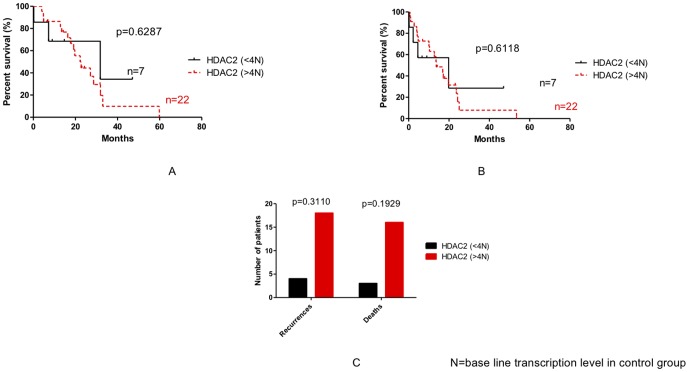
Impact of HDAC2 expression on the overall survival (A), the disease free survival (B) and recurrences and death in patients with pancreatic adenocarcinoma (C). N: base line transcription level in CG.

**Figure 10:**
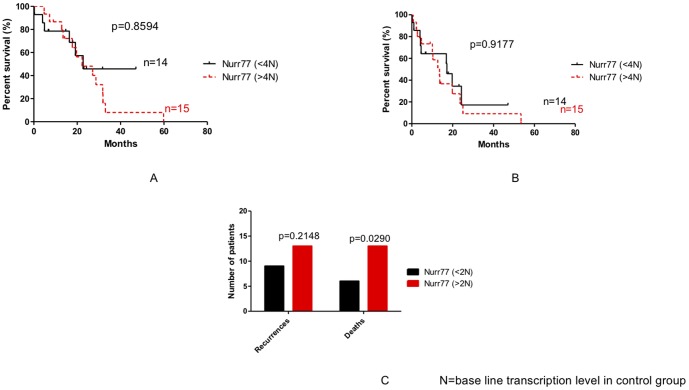
Impact of Nurr77 expression on the recurrences the overall survival (A), the disease free survival (B) and recurrences and death in patients with pancreatic adenocarcinoma (C). N: base line transcription level in CG.

We then examined the possible relationship of gene transcription levels (*HDAC7*, *HDAC2* and *Nurr77*) and the outcome of the disease. As shown in Figures 7, 8, 9 and 10, no statistically significant difference could be seen between groups in terms of overall and disease free survival when considering individuals expressing more of less than 4 times the baseline gene transcription level. However, when we analyzed the number of death and recurrences at the end of follow up, number of death and recurrences were significantly greater in patient with overexpression *HDAC7* (4 times the baseline gene transcription level) (Figure 8).

### Development of stable human pancreatic cell lines under-expressing or overexpressing HDAC7

**Figure 11:**
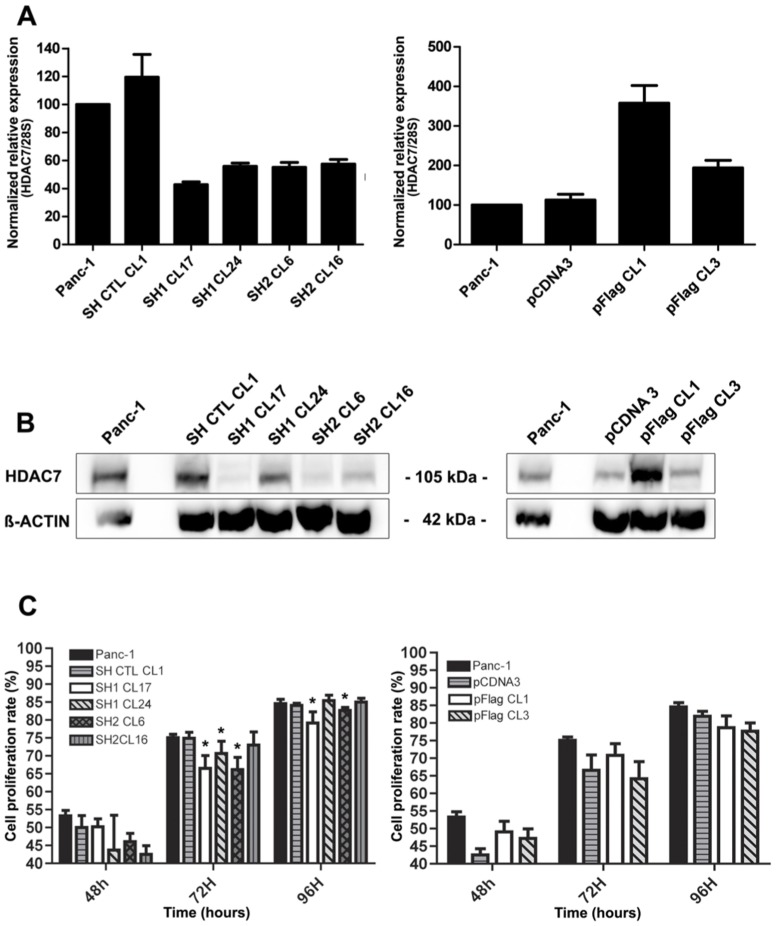
Expression of HDAC7 in transfected Panc-1 cell clones in relation to their growth capacity. Panc-1 cells were transfected with either Sure Silencing shRNA Plasmid for human HDAC7 (left panel) or pCDNA3-HDAC7 plasmid (right panel). Cell clones of each (SH1 CL17/SH1 CL24 and pFlag1/pFlag3, respectively) as well as control vectors (SH CTL and pCDNA3) were analyzed. Normalized relative expression of HDAC7/28S was assessed by Q RT-PCR (A), Relative expression was calculated using 28S as control gene and normalized to Panc-1 cells. HDAC7 synthesis was evaluated by Western blot (B). Proliferation of transfected Panc-1 cell clones was evaluated by monitoring their mitochondrial respiratory chain activity using MTT assay (C). Data are means ± SD of three independent experiments. Percent proliferation was calculated as follows: at 48 h : OD at 48 h - OD at 24h /OD at 48 h; at 72h : OD at 72 h- OD at 48h / OD at 72 h; at 96h OD at 96h-OD at 72h / OD at 96 h. OD, optic densitometry.

To assess the possible involvement of HDAC7 in cell proliferation capacity, we generated recombinant human Panc-1 tumoral cell clones using a set of four commercially available shRNA constructs and a corresponding control plasmid. Moreover, the pCDNA3-HDAC7-Flag plasmid was used to achieve overexpression of the HDAC7 protein. Transfections were also performed using a pCDNA3 empty vector as control. Transcription of HDAC encoding gene was analyzed by Q RT-PCR. As shown in Figure 11A, the use of shRNA knockdown approach resulted in a significant reduction of HDAC7 mRNA production in 4 obtained cell clones (SH1 Cl17, SH1 Cl24, SH2 Cl6 and SH2 Cl16) compared to Panc-1 shRNA control cell clone (SH CTL-Cl1), whereas overexpression of HDAC7 gene induced higher levels of HDAC7 mRNA synthesis in 2 cell clones (pFlag Cl1 and pFlag Cl3). Complementary experiments using Western blotting and anti-HDAC7 specific antibodies reacted with total extracts from different tumor cell populations were performed to examine whether the alteration of *HDAC7* gene expression was associated with a modification of HDAC7 protein synthesis. Estimation of HDAC7expression indicated that the recombinant cell clones, contained approximately 20 to 80% less HDAC7 protein than SH CTL-Cl1 control cell clone. Moreover, the estimated ratio of HDAC7 synthesis in Panc-1 tumor cells transformed with the control vector (pCDNA3) versus those that overexpress HDAC7 was approximately 1 to 300% (Figure 11B). ß-actin protein was used as internal control.

### Growth rates of transfected Panc-1cell clones

As a first step, we determined the growth rates of the cell clones. The proliferation of Panc-1 parental cells, shRNA, shControl, HDAC7-FLAG and pCDNA3 transformed tumor cell clones measured over 96 h of culture is shown in Figure 11C. Interestingly, the rate of growth was significantly reduced in the case of 3 out of four clone cells carrying shRNA construct targeting HDAC7 encoding gene when compared to the parental Panc-1 tumor cells (p = 0.0015) at 48 h, and at 96 h of culture still two out of four clones had significant reduction of cell growth (p = 0.0021). Moreover, overexpression of HDAC7 did not significantly modify the growth capacity of parental cells over the time period of culture (p = 0.3161) suggesting therefore that additional copies of HDAC7 encoding gene are not necessary to reach optimal growth for the parental tumor Panc-1 cells.

## Discussion

To improve modern cancer therapy, there is an ongoing interest to identify signaling pathways and genes that might play a key role in carcinogenesis and the development of resistance to anti-tumor drugs. Since HDAC interacts with various molecular mechanisms implicated in gene expression, they have captured the attention of a large number of researchers. As a family of transcriptional co-repressors they have emerged as important regulators of cell differentiation, cell cycle progression and apoptosis [23]. The dysfunction of transcriptional repression mediated by HDACs may lead to carcinogenesis. Indeed, alteration of HDACs/SIRTs expression levels (overexpression and/or downregulation) has been observed in various types of cancer [24].

Eighteen mammalian HDACs have been characterized and grouped into four distinct classes: Class I includes HDACs 1, 2, 3 and 8; and Class II, further subdivided in IIa (HDACs 4, 5, 7 and 9) and IIb (HDAC6 and HDAC10). HDAC 11 shares conserved residues with Class I and II enzymes in their catalytic site and is allocated to Class IV [24]. Based on their primary structure, the SIR2 family [Hst proteins (Homologous of Sir two)] or sirtuins are currently grouped into five different classes [25]: Class I (Human SIRT1, 2, 3); Class II (SIRT4); Class III (SIRT5) and Class IV (SIRT6, 7). SIR-T8, which was characterized for the first time in thyroid carcinoma cell lines and tissue samples [7], was included in a Class IV.

The prognosis for patients with pancreatic adenocarcinoma is extremely poor with 5-year survival of less than 5% [26]. Therefore, a number of studies have been devoted to the analysis of the genetic alterations with the hope to identify putative biomarkers and/or therapeutic targets [27]. Using molecular approaches, a large set of genes has been shown to be overexpressed in pancreatic cancer [12], [28], [29]. Moreover, biocomputational tools allowed to demonstrate that among the most differentially expressed genes in pancreatic cancer were *Mesothelin*, *Muc4*, *Muc5A/C*, *Kallikrein 10*, *Transglutaminase 2*, *Fascin*, *TMPRSS3* and *Stratifin* [30].

Moreover, alterations in several key genes including those playing a role in the control of cell cycle (*K-ras*, *p53*, *p16^INK4a^*, and *Smad4*) have also been reported [31]. Furthermore, high nuclear localization of S100A6, a low molecular weight calcium binding protein that belongs to S100 family of proteins, is significantly associated with poor survival in pancreatic cancer patients [32]. Epigenetic modifications such as changes in histone acetylation or methylation which influence the chromatin accessibility and gene expression have also been involved in altered critical genes expression in pancreatic cancer [33].

Given that a number of studies have shown that HDACs/SIRTs are among key factors that control gene expression, we have explored in a preliminary report their expression levels in a relatively few cases of pancreatic surgically resected pancreatic tissues and found that 9 out of 11 PA samples displayed increased expression of *HDAC7* mRNA transcripts [15]. The present study was conducted using increased number of pancreatic malignant as well as normal pancreatic tissues in order to explore more accurately the HDACs/SIRTs gene expression. The data revealed that most of the PA tumors analyzed (25/29, 86%) showed increased expression of HDAC7 encoding gene when compared to CP and B tumor samples, in agreement with our previous observations. Moreover, upregulation of *HDAC2*, an observation already reported by independent investigators [9], [10] has also been evidenced in our study population of PA.

In the present study, by using a new approach we provide evidence that several genes namely HDAC7, HDAC2 and Nur77 are overexpressed in significantly high percentage of pancreatic adenocarcinoma tumors compared to benign tumors and chronic pancreatitis. The most prominent gene overexpression levels been observed for HDAC7 encoding gene, in agreement with our previous preliminary observations. Furthermore, qPCR-based approach revealed high *Nur77* transcript levels associated with most of the PA tumors, an observation not anticipated, as this link, to our knowledge, has not been reported for PA tumors. Furthermore the quantitative method of HDAC7 and Nurr77 immunostaining clearly demonstrate that increased expression of both genes is associated with adenocarcinomas of the pancreas. The possible relationship between HDAC7, Nurr77, HDAC2 and the outcome of the disease was examined. Number of recurrences were significantly greater in patients with an overexpression of HDAC7. Recent study demonstrated that HDAC7 silencing by siRNA was unable to decrease cell growth in BxPC-3 cell lines [34]. This conflicting result could be explained by the different characteristics of the cell lines. For example, k-ras was mutated in Panc-1 and not in BxPC-3 [34]. Moreover, HDAC7 silencing in our study was obtained by shRNA.

Given the previous studies demonstrating the crucial role of HDAC7 as regulator in the thymocyte negative selection process through the down-regulating of the *Nur77* gene expression, an orphan nuclear receptor involved in antigen-induced apoptosis of thymocytes [35], we though it would be interesting to determine the pattern of *Nur77* gene expression simultaneously with those encoding *HDAC* and *SIRTs* in pancreatic tumor tissues. Although Nur77 affects cell proliferation and apoptosis through its capacity to bind to a variety of response elements leading to the regulation of their transactivation activities, the intrinsic function of Nur77 is not yet fully understood. The role of Nur77 as a positive regulator for apoptosis has been previously reported [36]. The authors found that in lung cancer cells, Nur77 overexpression is associated with retinoic acid (RA) resistance, and may contribute to cell proliferation and neoplastic transformation by blocking the inhibitory effect of RA on cell growth. Consistent with observations in this study, Yin *et al*. [37] also reported that Nur77 caused a delayed apoptotic process in lung cancer cells. In gastric cancer cells, translocation of Nur77 from the nucleus to the mitochondria and the subsequent Cyt c release from the mitochondria to the cytosol are required for tetradecanoylphorbol-1,3-acetate (TPA) to induce apoptosis. All-*trans* retinoic acid (ATRA) does not induce apoptosis in BGC-823 cells (gastric cancer cell line) in accordance with its failure of inducing translocation of Nur77 [36]. However, Nur77 still exerts its function of cell growth inhibition in the nucleus for the cell cycle regulation [36]. Therefore, these studies, combined with the reports mentioned above, demonstrate the divergent functions of Nur77 in the regulation of cell proliferation and apoptosis. The biological significance of Nur77 gene overexpression and its relation to HDAC7 in PA await further investigations. It is likely that both genes could participate in the angionesis process by controlling transcription factors involved in vascular gene expression. Indeed, it has been reported that HDAC7 is key modulator of endothelial cell migration and angiogenesis and regulate PDGF-Bp/PDGF-beta gene expression [38]. It is difficult to determine at this stage whether upregulation of HDAC7/HDAC2/Nur77 in pancreatic adenocarcinomas is a cause or a consequence of malignant progression. It is likely that the HDAC7 in pancreatic cancer could use the VEGF-PKD-HDAC7 axis in the settings of vascular disorders and could explain the potential metastatic of pancreatic cancer.

With more investigations, the involvement of HDAC7/HDAC2/Nur77 in the pathogenesis of pancreatic tumors can be clarified. It might lead to the appropriate utilization of the HDAC7/HDAC2/Nur77 as adjunctive markers for malignancy in pancreatic cancer, and towards the development of new approaches in the design of anti-pancreatic cancer therapy.

## Supporting Information

File S1 **Agreement reference of CRO2 for tissue collection.**
(PDF)

File S2 **Government attestation.**
(PDF)

Table S1 **Characteristics of patients with pancreatic adenocarcinoma.**
(DOCX)

Table S2 **Specific primers used in RT-PCR.**
(DOC)

Table S3 **Values of mean of** ***Cp*** **from the control group.**
(DOCX)
