# Supplementary material for: Further Characterization of HDAC and SIRT Gene Expression Patterns in Pancreatic Cancer and Their Relation to Disease Outcome
Source: PLoS One. 2014 Oct 2;9(10):e108520. doi: 10.1371/journal.pone.0108520 (PMC4183483; doi:10.1371/journal.pone.0108520)
Supplement: File S1 — Agreement reference of CRO2 for tissue collection. (PDF) [file pone.0108520.s001.pdf]

Comité de Protection des Personnes
Sud-Méditerranée II

Vincent PRADEL *Président*
Jean Pierre VIDAL *Vice-président*
Bertrand DUSSOL *Secrétaire Général*

Membres titulaires & *suppléants*

Collège technique

- Personnes qualifiées en recherche
  - Jean-Louis BERNARD
  - Pierre-Henri ROLLAND
  - Vincent PRADEL (méthodologiste)
  - Houtin BAGHDADI
  - Laurent *BOYER (méthodologiste)*
  - *Bertrand DUSSOL*
  - *Zo RAKOTONIAINA*
- Médecins généralistes
  - Claude SICHEL
  - *Pierre REYES*
- Pharmaciens hospitaliers
  - Nathalie SALES-AUSIAS
  - *Stéphane HONORE*
- Infirmières
  - Marie RAFFRAY
  - *Patrick BOANICHE*

Collège social

- Personnes qualifiées en éthique
  - Dominique TAILLEFER
  - *Michel CAILLOL*
- Psychologues
  - Janine RICOEUR
  - *Frédérique VINCENT*
- Travailleurs sociaux
  - Gilbert NAURAYE
  - *Isabelle LABAT*
- Juristes
  - Jean-Pierre VIDAL
  - Delphine BOHBOT
  - Marc André CECCALDI
  - *Olivier SCHWEITZER*
- Représentants d'associations et usagers
  - Patrick D'ANGIO
  - Jean ACCIARO
  - René *HULIN*
  - *Marie-Hélène ILTIS*

AVIS

Le Comité de Protection des Personnes Sud-Méditerranée II, agréé par arrêté ministériel en date du 31 mai 2012, constitué selon l'arrêté du Directeur Général de l'Agence Régionale de Santé de la région Provence Alpes Côte d'Azur en date du 13 juin 2012,

➢ en application des dispositions du Code de la Santé Publique et de la réglementation en vigueur applicables à la préparation, la conservation et l'utilisation des tissus, des cellules et de leurs dérivés,

➢ ayant été saisi par courrier du 11/06/2013 par l'INSERM PACA ET CORSE, d'un dossier de :

déclaration des organismes assurant pour les besoins de leurs propres programmes de recherche la conservation et la préparation, de tissus ou de cellules issus du corps humain et de leurs dérivés, des organes, du sang, de ses composants ou de ses produits dérivés, y compris la constitution et l'utilisation de collection d'échantillons biologiques humains (articles L1243-3 et R1243-49 et suivants du Code de la Santé Publique)

identifié par le Ministère de l' Enseignement Supérieur et de la Recherche sous la référence **DC-2013-1857** et dont le responsable scientifique est le **M. Dominique LOMBARDO**

en vue d'une demande d'avis portant sur une collection intitulée :

- FORMATION DE RECHERCHE **UMR-911 CR02**

➢

➢ ayant reçu ce dossier le 13/06/2013 et l'ayant enregistré sous la référence interne **213 C02,**

CPP Sud Méditerranée II - Hôpital Sainte Marguerite - 270 Boulevard Sainte Marguerite 13274 MARSEILLE Cédex 09
Identification SIRET : 130 016 116 00019 / Téléphone : (33) 491 745 609 - 0491 263 042 — *Télécopie :* (33) 491 745 616
Courriel : secretaires@cpp-sudmed2.fr — www.cpp-sudmed2.fr

- ayant examiné le dossier de cette collection lors de sa séance plénière du **05/07/2013** au cours de laquelle

L. BOYER, V.PRADEL, B. DUSSOL, C. SICHEL, M. CAILLOL,F. VINCENT, G. NAURAYE,JP. VIDAL, O. SCHWEITZER, J. ACCIARO,

le quorum général étant constaté,

après avoir entendu le rapporteur du collège scientifique et le rapporteur du collège social ont délibéré,

a émis un

AVIS FAVORABLE

à la constitution de cette collection, considérant que les conditions de validité, définies dans l'article R 1243-53 du Code de la Santé Publique, étaient réunies.

Le Secrétaire Général — Le Président

Pr Bertrand DUSSOL — Dr Vincent PRADEL

P/o
