# Supplementary material for: Further Characterization of HDAC and SIRT Gene Expression Patterns in Pancreatic Cancer and Their Relation to Disease Outcome
Source: PLoS One. 2014 Oct 2;9(10):e108520. doi: 10.1371/journal.pone.0108520 (PMC4183483; doi:10.1371/journal.pone.0108520)
Supplement: File S2 — Government attestation. (PDF) [file pone.0108520.s002.pdf]

Liberté • Égalité • Fraternité
RÉPUBLIQUE FRANÇAISE

MINISTÈRE
DE L'ENSEIGNEMENT SUPÉRIEUR
ET DE LA RECHERCHE

Paris le 05/07/2013

**Direction générale pour la recherche et l'innovation**

**Cellule bioéthique**

Affaire suivie par
Maryse Badji
Téléphone
01 55 55 86 68

Mél.
Gestion.conservation
@recherche.gouv.fr

1 rue Descartes
75231 Paris cedex 05

Le ministère de l'enseignement supérieur
et de la recherche

à

Monsieur le Délégué régional
INSERM DR Provence Alpes Côte d'Azur et Corse
BP 172
13276 MARSEILLE Cedex 9

**Objet :** Dossier de déclaration de conservation et préparation à des fins scientifiques d'éléments du corps humain (Responsable scientifique : Dominique LOMBARDO)

Conformément aux dispositions des articles R. 1243-49 et suivants du code de la santé publique, vous avez adressé à la ministre de l'enseignement supérieur et de la recherche un dossier de déclaration d'activité de conservation et de préparation d'éléments du corps humain pour les besoins des programmes de recherche de l'organisme que vous représentez.

La direction générale pour la recherche et l'innovation du ministère (Cellule de bioéthique) accuse réception de votre dossier, enregistré sous le n° DC-2013-1857.

La cellule s'est assurée que le comité de protection des personnes compétent a bien reçu le dossier.

En l'absence de décision expresse d'opposition du ministre de l'enseignement supérieur et de la recherche, l'activité déclarée peut commencer à l'issue d'un délai de trois mois à compter de la date de cet accusé de réception. Toutefois, ce délai peut-être suspendu s'il est demandé de fournir des informations manquantes ou incomplètes ou des compléments nécessaires à l'expertise du dossier.

Estelle ECHARD

---

Une attestation d'acceptation de votre déclaration peut, en cas de besoin, vous être délivrée à votre demande.

REÇU LE
08 AOUT 2013
Inserm Délégation Régionale PACA et Corse
