# Supplementary material for: Further Characterization of HDAC and SIRT Gene Expression Patterns in Pancreatic Cancer and Their Relation to Disease Outcome
Source: PLoS One. 2014 Oct 2;9(10):e108520. doi: 10.1371/journal.pone.0108520 (PMC4183483; doi:10.1371/journal.pone.0108520)
Supplement: Table S1 — Characteristics of patients with pancreatic adenocarcinoma. (DOCX) [file pone.0108520.s003.docx]

**Table S1.** Characteristics of patients with pancreatic adenocarcinoma; PD pancreaticoduodenectomy, LF left pancreatectomy ; AJCC CLASSIFICATION; tumor grade differentiation G x,1,2,3,4

| Patients | Surgery | Size of tumor  (mm) | Nodes resected | Nodes envaded | T | N | Metastases* | Stage | Retroperitoneal margin | | | | Perinervous invasion | Vascular embolous | Lymphatic  Embolus | Differentiation (G0) | Level HDAC7expression/normal |
| --- | --- | --- | --- | --- | --- | --- | --- | --- | --- | --- | --- | --- | --- | --- | --- | --- | --- |
|  |  |  |  |  |  |  |  |  | R* | >1  mm | 0  mm | 0-1mm |  |  |  |  |  |
| 1 | PD | 45 | 12 | 4 | 2 | 1 | 0 | 2B | 0 | yes | 0 | 0 | yes | 0 | 0 | 2 | 4.5 |
| 2 | LP | 47 | 5 | 1 | 3 | 1 | yes | 4 | 0 | yes | 0 | 0 | yes | yes | yes | 2 | 21.7 |
| 3 | PD | 30 | 12 | 2 | 2 | 1 | 0 | 2B | 0 | yes | 0 | 0 | yes | yes | yes | 2 | 5.4 |
| 4 | PD | 30 | 15 | 4 | 3 | 1 | 0 | 4 | 0 | yes | 0 | 0 | yes | yes | yes | 2 | 4.9 |
| 5 | PD | 18 | 6 | 2 | 3 | 1 | 0 | 2B | yes | 0 | yes | 0 | 0 | 0 | 0 | 2 | 3271.8 |
| 6 | LP | 35 | 6 | 0 | 3 | 0 | 0 | 2A | 0 | yes | 0 | 0 | yes | yes | yes | 1 | 20.3 |
| 7 | PD | 60 | 4 | 0 | 3 | 0 | 0 | 2A | 0 | yes | 0 | 0 | 0 | 0 | 0 | 2 | 13.1 |
| 8 | PD | 25 | 42 | 1 | 3 | 1 | 0 | 2B | 0 | yes | 0 | 0 | yes | yes | yes | 1 | 1.5 |
| 9 | PD | 45 | 13 | 2 | 3 | 1 | 0 | 2B | 0 | yes | 0 | 0 | yes | yes | yes | 1 | 157,6 |
| 10 | PD | 25 | 10 | 0 | 3 | 0 | 0 | 2A | 0 | yes | 0 | 0 | 0 | 0 | 0 | 1 | 0.7 |
| 11 | PD | 23 | 11 | 3 | 3 | 1 | 0 | 2B | yes | 0 | yes | 0 | yes | yes | yes | 2 | 3.1 |
| 12 | LP | 25 | 10 | 0 | 3 | 0 | 0 | 2A | 0 | yes | 0 | 0 | yes | yes | yes | 1 | 2.8 |
| 13 | PD | 20 | 1 | 0 | 2 | 0 | 0 | 1B | 0 | yes | 0 | 0 | 0 | 0 | 0 | 1 | 0.3 |
| 14 | PD | 20 | 1 | 0 | 3 | 0 | 0 | 2A | 0 | yes | 0 | 0 | yes | yes | yes | 1 | 13.4 |
| 15 | PD | 30 | 10 | 10 | 3 | 0 | 0 | 2A | 0 | yes | 0 | 0 | yes | 0 | 0 | 1 | 6.8 |
| 16 | PD | 15 | 4 | 0 | 3 | 0 | 0 | 2A | 0 | yes | 0 | 0 | yes | 0 | 0 | 1 | 0.3 |
| 17 | PD | 52 | 29 | 6 | 3 | 1 | 0 | 2B | yes | 0 | 0 | yes | yes | 0 | 0 | 1 | 9.4 |
| 18 | PD | 30 | 9 | 0 | 3 | 0 | 0 | 2A | 0 | yes | 0 | 0 | yes | yes | yes | 1 | 873.8 |
| 19 | LP | 20 | 7 | 1 | 3 | 1 | 0 | 2B | yes | 0 | yes | 0 | yes | yes | yes | 1 | 1.0 |
| 20 | LP | 43 | 11 | 1 | 4 | 1 | yes | 4 | 0 | yes | 0 | 0 | yes | yes | yes | 3 | 99.1 |
| 21 | PD | 60 | 5 | 1 | 3 | 1 | 0 | 2B | 0 | yes | 0 | 0 | yes | yes | yes | 1 | 22.7 |
| 22 | PD | 20 | 11 | 0 | 3 | 0 | 0 | 2A | 0 | yes | 0 | 0 | 0 | 0 | 0 | 3 | 618.2 |
| 23 | PD | 13 | 12 | 4 | 3 | 1 | 0 | 2B | 0 | yes | 0 | 0 | yes | 0 | 0 | 1 | 12.8 |
| 24 | PD | 23 | 9 | 4 | 3 | 1 | 0 | 2B | 0 | yes | 0 | 0 | yes | yes | yes | 2 | 25.2 |
| 25 | PD | 15 | 34 | 0 | 2 | 0 | 0 | 1B | yes | 0 | yes | 0 | yes | yes | yes | 2 | 3228.2 |
| 26 | PD | 35 | 31 | 4 | 3 | 1 | 0 | 2B | yes | 0 | 0 | yes | 0 | 0 | 0 | 1 | 8.5 |
| 27 | LP | 30 | 14 | 0 | 2 | 0 | 0 | 1B | 0 | yes | 0 | 0 | 0 | 0 | 0 | 1 | 6.1 |
| 28 | PD | 3 | 12 | 1 | 3 | 1 | 0 | 2B | yes | 0 | yes | 0 | yes | 0 | 0 | 1 | 3.6 |
| 29 | LP | 3,5 | 5 | 0 | 3 | 0 | 0 | 2A | 0 | yes | 0 | 0 | yes | 0 | 0 | 2 | 7.2 |
