# Supplementary material for: Further Characterization of HDAC and SIRT Gene Expression Patterns in Pancreatic Cancer and Their Relation to Disease Outcome
Source: PLoS One. 2014 Oct 2;9(10):e108520. doi: 10.1371/journal.pone.0108520 (PMC4183483; doi:10.1371/journal.pone.0108520)
Supplement: Table S2 — Specific primers used in RT-PCR. (DOC) [file pone.0108520.s004.doc]

Table S2: Specific primers used in RT-PCR

| Primer | Direction | Sequence (5’ to 3’) |
| --- | --- | --- |
| HDAC1 | Forward | GTC CAG ATA ACA TGT CGG AGT ACA GC |
| HDAC1 | Reverse | CGA TGT CCG TCT GCT GCT TAT TAA G |
| HDAC2 | Forward | CCT CAT AGA ATC CGC ATG ACC CAT AAC |
| HDAC2 | Reverse | AGA CAT GTT ATC TGG TCT TAT TGA CCG TAG |
| HDAC3 | Forward | CAA GCC ATA CCA GGC CTC CCA GC |
| HDAC3 | Reverse | GAG ATG CGC CTG TGT AAC GCG AG |
| HDAC4 | Forward | CCT GCA CAG ACA CGG GGA AGG TG |
| HDAC4 | Reverse | GAG CTG CTC TTC AGA CAG CAA GC |
| HDAC7 | Forward | GTC CAG GTG ATC AAG AGG TCA |
| HDAC7 | Reverse | AAC ACC TGA GGG TGC TGC T |
| SIRT1 | Forward | TGC GGG AAT CCA AAG GAT AAT TCA GTG TC |
| SIRT1 | Reverse | CTT CAT CTT TGT CAT ACT TCA TGG CTC TAT G |
| SIRT2 | Forward | CAG AAC ATA GAT ACC CTG GAG CGA A |
| SIRT2 | Reverse | AAG GTC CTC CAG CTC CTT CTT C |
| SIRT3 | Forward | GTC GGG CAT CCC TGC CTC AAA GC |
| SIRT3 | Reverse | GGA ACC CTG TCT GCC ATC ACG TCA G |
| SIRT5 | Forward | CGA GTC GTG GTC ATC ACC CAG AAC ATC |
| SIRT5 | Reverse | ACT CTT GTA ATT CTC AGC CAC AAC TCC AC |
| SIRT6 | Forward | GAG GAG CTG GAG CGG AAG GTG TG |
| SIRT6 | Reverse | GGC CAG ACC TCG CTC CTC CAT GG |
| NUR77 | Forward | GCT GCA GAA TGA CTC CAC C |
| NUR77 | Reverse | ACA GCA GCA CTG GGC TTA |
| 28S | Forward | AGC CGA TCC ATC ATC CGC AAT G |
| 28S | Reverse | CAG CCA AGC TCA GCG CAA C |
