# Supplementary material for: Further Characterization of HDAC and SIRT Gene Expression Patterns in Pancreatic Cancer and Their Relation to Disease Outcome
Source: PLoS One. 2014 Oct 2;9(10):e108520. doi: 10.1371/journal.pone.0108520 (PMC4183483; doi:10.1371/journal.pone.0108520)
Supplement: Table S3 — Values of mean of Cp from the control group. (DOCX) [file pone.0108520.s005.docx]

**Table S3.** Values of mean of *Cp* from the control group. Comparisons were made by Wilcoxon testand differences were considered significant at *p*< 0.05; F female, M male.

| Gender | Tissue samples | Age (years) | Genes transcripts Ct (Mean Cp* ) | | | | | | | | | | | |
| --- | --- | --- | --- | --- | --- | --- | --- | --- | --- | --- | --- | --- | --- | --- |
|  |  |  | 28S | HDAC1 | HDAC 2 | HDAC 3 | HDAC 4 | HDAC 7 | SIRT1 | SIRT2 | SIRT3 | SIRT5 | SIRT6 | Nur77 |
| F | BD-1 | 80 | 25.45 | 28.83 | 36.94 | 37.52 | 33.94 | 45.22 | 36.97 | 35.93 | 38.20 | 32.98 | 31.32 | 25.57 |
| F | NP-1 | 53 | 23.29 | 31.95 | 33.70 | 32.41 | 39.39 | 39.68 | 32.72 | 32.99 | 31.30 | 32.82 | 31.90 | 25.68 |
| M | NP-2 | 3 | 19.70 | 28.93 | 29.60 | 27.15 | 34.14 | 38.99 | 31.65 | 32.91 | 30.00 | 31.75 | 30.37 | 25.50 |
| M | NP-3 | 44 | 18.64 | 25.91 | 28.57 | 26.89 | 30.93 | 38.27 | 30.89 | 36.19 | 30.53 | 26.41 | 27.60 | 21.25 |
| M | NP-4 | 23 | 18.16 | 24.74 | 27.80 | 25.75 | 30.10 | 36.46 | 30.92 | 35.91 | 24.20 | 26.73 | 26.85 | 23.01 |
| F | G-A | 52 | 16.55 | 22.46 | 24.30 | 27.73 | 29.75 | 35.00 | 29.22 | 35.21 | 30.06 | 25.96 | 28.24 | 22.15 |
| F | AP-1 | 73 | 22.41 | 30.51 | 34.35 | 36.26 | 35.10 | 41.26 | 27.82 | 35.76 | 39.09 | 32.18 | 31.27 | 29.43 |
| F | AP-2 | 60 | 20.41 | 26.68 | 30.47 | 25.64 | 28.65 | 35.58 | 32.33 | 33.87 | 31.92 | 28.75 | 28.06 | 25.41 |
| F | gastrinoma | 65 | 20.67 | 27.55 | 29.59 | 26.14 | 29.10 | 36.76 | 33.70 | 33.78 | 34.50 | 30.16 | 29.24 | 25.54 |
| M | AP-3 | 71 | 23.73 | 31.89 | 31.87 | 31.40 | 37.44 | 37.91 | 38.78 | 34.30 | 34.21 | 34.85 | 35.95 | 27.07 |
| M | BD-2 | 64 | 20.87 | 25.93 | 28.66 | 26.90 | 29.88 | 39.18 | 32.97 | 36.05 | 29.77 | 31.94 | 27.65 | 24.76 |
|  |  | *p* values | 0.2568 | 0.8501 | 0.5708 | 0.5708 | 0.7055 | 1.0000 | 0.2568 | 0.5708 | 0.1306 | 0.5708 | 0.5708 | 0.1859 |
